# Proteome remodeling upon disruption of virulence-associated CipC from *Cryptococcus neoformans*

**DOI:** 10.1128/mra.01138-24

**Published:** 2025-03-06

**Authors:** Wan Yun Zhu, Lauren Segeren, Norris Chan, Brianna Ball, Jennifer Geddes-McAlister

**Affiliations:** 1 Molecular and Cellular Biology Department, University Of Guelph 317113 , Guelph, Ontario, Canada; University of Notre Dame, Notre Dame, Indiana, USA

**Keywords:** proteomics, virulence, *Cryptococcus neoformans*

## Abstract

The human fungal pathogen, *Cryptococcus neoformans*, produces an array of virulence factors that regulate the production of a polysaccharide capsule and melanin, thermotolerance, and the release of extracellular enzymes. Here, we applied quantitative proteomics to investigate the role of CipC, a known virulence-associated protein, in the fungus.

## ANNOUNCEMENT

*Cryptococcus neoformans* is a human fungal pathogen found ubiquitously within the environment and associated with infection of primarily immunocompromised individuals (1–3). Without the activation of an effective immune response, the pathogen can survive, proliferate, and disseminate throughout the host through the action of diverse virulence factors (4). These virulence factors include a polysaccharide capsule to protect the fungus from phagocytosis by macrophages, melanin production to neutralize reactive oxygen species, thermotolerance to survive at human physiological temperatures, and extracellular enzymes for host tissue degradation and invasion (5–8). We previously used mass spectrometry-based proteomics to explore the production of fungal virulence factors during infection using *in vitro* (macrophages) and *in vivo* (murine) models of disease (9–12). Based on our studies, we investigated the proteome response of *C. neoformans* upon disruption of CipC, a virulence-associated fungal protein (13, 14).

In this study, we cultured the *C. neoformans* wild-type H99 strain (ATCC 208821) on yeast peptone dextrose (YPD) agar plates with incubation overnight at 30°C. The H99 strain was used to generate a gene deletion strain of *cipC* via techniques (i.e., biolistic transformation, Southern blot) we previously described (11, 12). For culturing, a single colony (three biological replicates) of the WT and *cipC*∆ strains were transferred to YPD broth (5 mL) and incubated at 30°C with shaking (200 rpm) overnight followed by a 1:100 dilution into 5 mL of yeast nitrogen base (YNB) broth. Cultures were collected (1 mL) at mid-log phase (∼8 h) by centrifugation at 3,500 rpm for 5 min and washing the cell pellets twice with phosphate-buffered saline (PBS, pH 7.4). For protein extraction, pellets were prepared as we previously described (15) by resuspending cells in cold 100 mM Tris-HCl (pH 8.5) containing 2% sodium dodecyl sulfate, probe sonicating (30% power, 30 s on/30 s off, ice-bath), and adding dithiothreitol (10 mM final) at 95°C for 10 min and iodoacetamide (5.5 mM final) for 20 min at room temperature in the dark. Proteins were precipitated overnight with the addition of 100% ice-cold acetone (−20°C) followed by washing (80% acetone), resuspension in 8 M urea/40 mM HEPES, and quantification (16). Proteins were digested overnight at room temperature with trypsin/LysC (1:50 enzyme:protein), and peptides were desalted with STop And Go Extraction (STAGE) tips (17).

Peptides were resuspended in buffer A (0.1% trifluoracetic acid) separated on a 2 h gradient at 250 nL/min flow rate with buffer B (80% acetonitrile, 0.5% acetic acid) using a nanoflow liquid chromatography (Neo VanQuish; Thermo Fisher Scientific) system with a 50 cm PepMap spray column (75 µm inner diameter) filled with 2 µm C18 reverse-phase silica beads (Thermo Fisher Scientific). Electrospray ionization of peptides was coupled with an Orbitrap Exploris 240 hybrid quadrupole-orbitrap mass spectrometer (Thermo Fisher Scientific) operated in data-dependent acquisition mode with full scans (*m/z* 400 to 2,000) acquired at 120,000 resolution. Raw mass spectrometry data files were processed with MaxQuant (version 2.4.10.0) (18) powered by Andromeda (19) against the *C. neoformans* var. *grubii* serotype A (strain H99/ATCC 208821) FASTA (7,429 sequences; 11 October 2024) from UniProt (20). MaxQuant parameters were set as default except for a minimum number of two peptides per protein, label-free quantification by 1 ratio, and match between runs enabled. Data were analyzed using Perseus (version 1.6.2.2) (21). Proteins were filtered (contaminants, reverse peptides, peptides identified by site, valid values of 2 of 3 replicates in at least one group), log_2_ transformed, normalized (subtraction of median from each sample), and imputed (normal distribution with a width of 0.3 and a downshift of 1.8). Statistical testing was performed using a Student’s *t*-test *P*-value < 0.05, a Benjamini-Hochberg false discovery rate correction factor of 0.05 (22), and *S*_0_ = 1.

**Fig 1:**
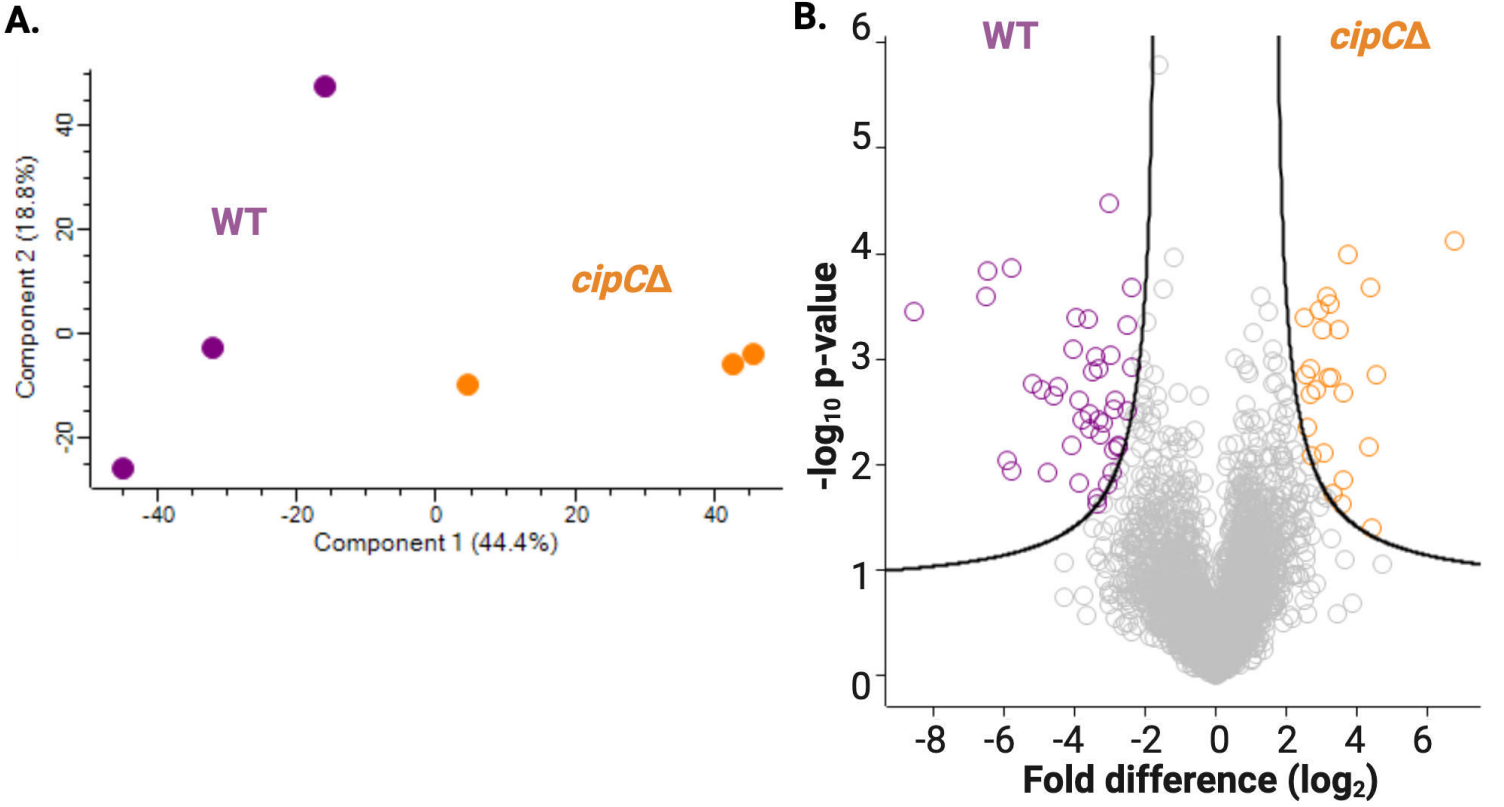
Proteome profiling of *C. neoformans cipC*∆. (**A**) Principal component analysis. (**B**) Volcano plot. Student’s *t*-test *P*-value < 0.05, FDR = 5%, *S*_0_ = 1. Performed in biological triplicate.

In the *C. neoformans* total proteome, we identified 3,347 proteins with replicate reproducibility of 87.9% for WT and 86.8% for *cipC*∆. By principal component analysis, we observed distinction between the strains (component 1, 44.4%) and replicates (component 2, 18.8%) (Fig. 1A). We also observed a significant change in the abundance of 66 proteins with 41 proteins showing a significant increase in the WT strain (including CipC, as a positive control) and 25 proteins showing a significant increase in the *cipC*∆, including a peptidase with proposed roles in fungal virulence (23–26) and transcriptional regulators. Overall, this study provides new biological insight into the roles of CipC in *C. neoformans* and proposes a new connection between fungal virulence and peptidase regulation.

## Data Availability

The mass spectrometry-based proteomics data (W# files: Wildtype; 7_# files: Mutant) is available through PRIDE Proteome Exchange with accession number PXD056761.
